# A White Paper on Advancing Long‐Acting Therapeutics for Maternal and Pediatric Health by Bridging Gaps in Clinical Research, Access and Regulation

**DOI:** 10.1002/cpt.70288

**Published:** 2026-04-14

**Authors:** Moherndran Archary, Robert Bies, Osei Boateng, Rachel Daley, Pierre Gashema, Mili Karina, Emily Njuguna, Sharon Nachman, Nathaniel Nkrumah, Carolyne Odula, Andrew Owen, Ethel D. Weld, Prajith Venkatasubramanian, Adeniyi Olagunju, the Community of Practice for Long‐acting Therapeutics for Maternal and Paediatric Health, Rana Abutaima, Dorothy Akongo, Abdulnaser Alsharaa, Moherndran Archary, Shakir Atoyebi, Benoit Bestgen, Robert Bies, Osei Boateng, Andrew Butler, Edmund Capparelli, Rachel Daley, Layla Davies, Joelle Dountio, Henry Enzama, Pierre Gashema, Katila George, Dan Hawcutt, Rene Holm, Patrick Gad Iradukunda, Elodie Jambert, Mili Karina, Linda Lewis, Grace Miheso, Sebastien Morin, Andrew Morrison, Sharon Nachman, Shadia Nakalema, Emily Njunga, Nathaniel Nkrumah, Carolyne Odula‐Obonyo, Brenda Okware, Adeniyi Olagunju, Andrew Owen, Martina Penazzato, Natella Rakhmanina, Mphako Brighton Ratlabyana, Rachel Scott, Kimberly Struble, Andre‐Marie Tchouatieu, Catherine Unsworth, Prajith Venkatasubramanian, Catriona Waitt, Ethel Weld, Janine Winterbottom, Leena Zino

**Affiliations:** ^1^ Department of Paediatrics and Child Health, African Health Research Institute University of KwaZulu‐Natal, Victoria Mxenge Hospital Durban South Africa; ^2^ School of Pharmacy and Pharmaceutical Sciences State University of New York Buffalo New York USA; ^3^ OKB Hope Foundation Kumasi Ghana; ^4^ Department of Biochemistry, Cell and Systems Biology University of Liverpool Liverpool UK; ^5^ Centre of Excellence for Long‐acting Therapeutics University of Liverpool Liverpool UK; ^6^ Department of Research Repolicy Research Centre Kigali Rwanda; ^7^ Care for a Child's Heart Nairobi Kenya; ^8^ Africa Neonatal Association Kigali Rwanda; ^9^ PATH Nairobi Kenya; ^10^ Renaissance School of Medicine Stony Brook University Stony Brook New York USA; ^11^ Food and Drugs Authority Accra Ghana; ^12^ Department of Obstetrics and Gynaecology The University of Nairobi‐Health Services Nairobi Kenya; ^13^ Department of Pharmacology and Therapeutics University of Liverpool Liverpool UK; ^14^ Division of Clinical Pharmacology, Department of Medicine Johns Hopkins University School of Medicine Baltimore Maryland USA; ^15^ Division of Infectious Diseases, Department of Medicine Johns Hopkins University School of Medicine Baltimore Maryland USA

## Abstract

As use cases for long‐acting therapeutics expand across clinical indications, there is a critical need to ensure the inclusion of women who are pregnant or breastfeeding, infants and children—populations with a historical gap in the availability of interventions already approved for use in adults. This White Paper synthesizes insights from a special session during the 1st of July 2025 workshop of the Community of Practice for Long‐Acting Therapeutics for Maternal and Paediatric Health. It was hosted by the University of Liverpool Centre of Excellence for Long‐acting Therapeutics. Attendees included stakeholders drawn from clinical practice, patient advocacy groups, academia, pharmaceutical industry, regulatory agencies, product development partners, and public health organizations. Four focus groups—centered on maternal health, pediatric health, access, and regulation—addressed three key questions: (i) What are the most urgent gaps that could hinder the adoption of long‐acting therapeutics for maternal and pediatric health indications? (ii) What critical actions are needed to address these gaps? (iii) What partnerships must be initiated or strengthened to enable or accelerate these actions? Actionable strategies to accelerate progress were identified. Key themes that emerged from the discussion included the need for inclusive and context‐sensitive research designs, harmonized regulatory frameworks, culturally responsive implementation strategies, and sustainable funding mechanisms. Platforms for fostering interdisciplinary collaboration, amplifying diverse stakeholder voices, and promoting transparency in innovation are needed. Partnership models that support inclusive development and equitable deployment will be central to successful integration and to realize the full potential of long‐acting therapeutics in advancing maternal and pediatric health.

Long‐acting therapeutics represent a transformative advancement in healthcare delivery, offering new opportunities to facilitate better adherence, improve outcomes, and support better healthcare resource utilization for all populations.^1^ Sustained therapeutic drug concentrations over extended durations—from weeks with oral or transdermal products to several months with injectable or implantable systems—eliminate the necessity for daily lifelong pill intake.^2^ Globally, adherence to long‐term therapies in chronic conditions averages only 50%, contributing significantly to preventable morbidity, mortality, and rising healthcare costs.^3^ Long‐acting medicines offer a compelling solution to this challenge. For instance, long‐acting injectable (LAI) antiretroviral therapies (ART) for HIV have achieved high rates of viral suppression at 12 months—even among individuals with a history of poor adherence to daily oral regimens.^4^ Likewise, long‐acting contraceptive implants demonstrate continuation rates exceeding 90% after 1 year, surpassing short‐acting methods and highlighting the transformative potential of sustained‐release systems in improving health outcomes.^5^, ^6^ Hence, long‐acting therapeutics are not merely a promising strategy—they are increasingly regarded as a paradigm shift in the future of therapeutic interventions. This approach holds particular promise for maternal and pediatric health, where adherence challenges—such as nausea and vomiting during pregnancy or poor acceptability of unpalatable pediatric oral formulations—and the need for long‐term prophylaxis or treatment can be addressed through simplified, long‐acting delivery strategies.^7^, ^8^, ^9^ While long‐acting therapeutics offer clear advantages, they also present population‐specific concerns for pregnant and lactating women. These include the challenge of prolonged systemic exposure if adverse effects occur or if pregnancy is recognized later than expected in the case of drugs not recommended for use during pregnancy, and limited evidence on *in utero* fetal and infant exposure through breastmilk. These considerations highlight the need for dedicated pharmacokinetic and safety studies to guide the safe use of long‐acting formulations in pregnancy and lactation.

In view of their promise, there is growing interest in ensuring that no population is left behind in their development, adoption, and implementation.^10^ Of particular interest are children and women (The term “women” in this White Paper refers specifically to individuals assigned female at birth [i.e., cisgender females]) during pregnancy and breastfeeding, two populations that have historically been excluded from early stages of drug development clinical trials.^11^ This has been fostered by ethical and regulatory conservatism and over‐protectionism, resulting in persistent evidence gaps and widespread off‐license drug use.^12^, ^13^ The absence of pharmacokinetic and safety data delays regulatory approval and programmatic uptake, often by five to 7 years following adult authorization.^14^ Few clinical trials for long‐acting formulations globally include maternal or pediatric participants.^15^, ^16^ These delays risk perpetuating inequities in access and limit the potential of long‐acting therapeutics to improve maternal and child health globally, particularly in regions where health indices in these populations require improvement. For instance, the burden of infectious and chronic diseases remains disproportionately high in low‐ and middle‐income countries (LMICs).

Recognizing these challenges, the University of Liverpool Centre of Excellence for Long‐acting Therapeutics convened and hosted a workshop of the Community of Practice for Long‐acting Therapeutics in Maternal and Pediatric Health on the 1st of July 2025, with funding from Unitaid. The Community of Practice includes cross‐sector stakeholders from 17 countries across 5 continents (**Table** **S1**). Importantly, it is not organized around therapeutic area silos, but around cross‐cutting scientific, regulatory, and access challenges relevant to long‐acting therapeutics across maternal and pediatric health. The four sessions of the workshop explored pathways for advancing long‐acting therapeutics for maternal and pediatric health priorities (sessions 1 and 2), global health and regulatory considerations (session 3), and a cross‐cutting focus group discussion session on gap identification/blocking strategies (session 4). The discussions underscored the urgency of developing integrated maternal‐child research frameworks, inclusive trial designs, and community‐driven implementation strategies that align innovation with health equity. Participants further emphasized the need to address funding inequities, streamline regulatory pathways, and ensure that long‐acting innovations are not confined to high‐income countries but are equitably developed and deployed in regions with the highest disease burdens and the highest unmet need.^10^, ^17^ Insights from the other sessions are disseminated separately, including a review article on the maternal health session published in this issue.^18^

This manuscript translates insights from the workshop's focus group discussion (session 4) into a concrete roadmap for action. It aims to (i) identify key barriers impeding the development and deployment of long‐acting therapeutics for maternal and pediatric populations; (ii) regulatory and ethical frameworks that support inclusion and safety without compromising innovation; and (iii) outline scalable implementation models that strengthen access, affordability, and trust. By bridging the current gaps in access, regulation, and implementation, the global health community can ensure that the long‐acting therapeutic revolution delivers on its promise to make healthcare more equitable, sustainable, and lifesaving for mothers and children worldwide.

## METHODOLOGY

### Session design and discussion framework

Members of the Community of Practice who attended the workshop included stakeholders from diverse sectors. To balance expertise and avoid clustering of similar perspectives, a cross‐sector group of between 6 and 8 members was assigned to each thematic focus group using information on their profession, sector, and region. The focus group discussions were designed to enable focused dialogue on identifying priority gaps, solutions, and enabling partnerships.

Four focus groups—centered on maternal health, pediatric health, access, and regulation—addressed three key questions, adapted to the thematic focus of each group:

- What are the most urgent gaps that could hinder the adoption of long‐acting therapeutics for maternal and pediatric health indications? Participants examined the most pressing challenges that could hinder the adoption and impact of long‐acting therapeutics. This included research and knowledge gaps, regulatory bottlenecks, technological know‐how, equitable access, and disparities between LMICs and HICs.
- What critical actions are needed to address these gaps? Each group identified and discussed critical actions needed to bridge these gaps, including ethical inclusion in clinical trials, voluntary licensing, equitable access commitments, regulatory policy alignment, and practical implementation strategies.
- What partnerships must be initiated or strengthened to enable or accelerate these actions? Here participants explored the types of partnerships required to accelerate progress, including existing ones, and the potential convening and advocacy role of the Community of Practice.

Rapporteurs facilitated the discussions using a semi‐structured format, ensuring alignment with the guiding questions, encouraging reflection on own professional knowledge and experience, and insights from the plenary sessions.

### Documentation and integration

Discussions in each group were live captured using dictaphone for later transcription. Participants used sticky notes to document additional thoughts during the discussions. A high‐level summary of key points from each focus group was presented by the rapporteur during a plenary report‐back session. This provided opportunity for cross‐group learning and identification of overarching themes. The full audio transcript was used by the rapporteur to synthesize a concise description of key points and recommendations. Discussion of the feedback from each focus group during the report‐back session generated a set of actionable recommendations.

Insights from these discussions are presented below, starting with identified gaps, critical actions, and necessary enabling partnerships. Recommendations for consideration by all stakeholders involved in the development and implementation of long‐acting therapeutics for maternal and therapeutic health indications are then presented. These insights and recommendations will inform the activities of the Community of Practice and contribute to a shared roadmap for advancing this important area globally.

## OUTCOMES OF FOCUS GROUP DISCUSSIONS

### Maternal health

#### Existing gaps

**Figure 1:**
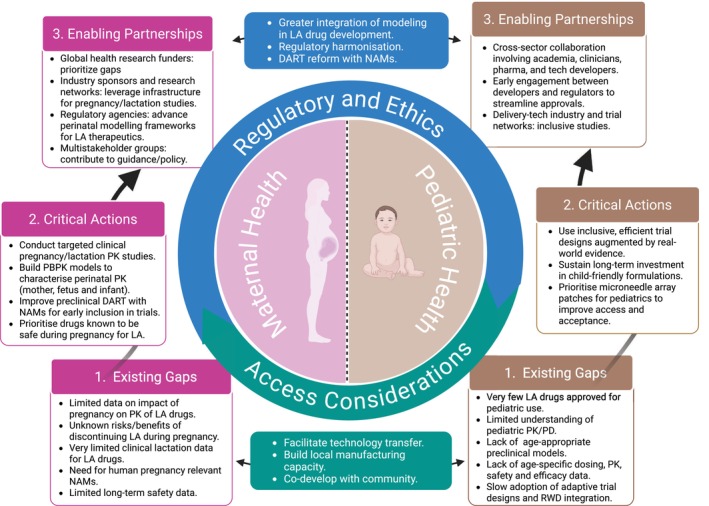
Existing gaps, critical actions, and enabling partnerships for advancing long‐acting therapeutics in maternal and pediatric health. Maternal and pediatric health are represented as core domains with regulatory, ethics, and access considerations functioning as cross‐cutting domains. The interdependence illustrated here highlights the need for coordinated, multisector approaches to accelerate development, evaluation, and equitable implementation of long‐acting therapeutics. Abbreviations: DART, developmental and reproductive toxicology; LA, long‐acting; NAMs, new approach methodologies; PBPK, physiologically based pharmacokinetic modeling; PD, pharmacodynamics; PK, pharmacokinetics; RWD, real‐world data.

Understanding of pregnancy‐induced alterations in the pharmacokinetics of long‐acting therapeutics remains limited—not only for recently approved agents such as LA‐ARVs, but also for long‐established treatments like long‐acting antipsychotics. In view of the well‐documented impact of physiological changes during pregnancy on daily oral medications pharmacokinetics,^19^ it is critical to confirm exposure adequacy throughout the dosing interval when long‐acting therapeutics are used during pregnancy (**Figure** **1**). Existing data on extended presence of long‐acting drugs in the system after discontinuation also raises questions about the necessity or even prudence of discontinuing long‐acting treatment during pregnancy.^20^ Clinical lactation studies are equally needed to characterize breastfed infant exposure to maternal long‐acting therapeutics, both in the context of active and discontinued treatment. Additionally, lack of human pregnancy‐specific safety data that limits the use of certain drugs available as daily oral formulations will likely similarly preclude the use of their long‐acting counterpart formulations.^16^ For new drugs that are primarily available in long‐acting forms, there are generally no comprehensive, long‐term trials in pregnant women to assess long‐term maternal and fetal safety.^21^ These gaps result partially from the use of animal models that often do not adequately recapitulate human physiology in preclinical developmental and reproductive toxicology studies. Beyond empirical data gaps, the modeling evidence base remains nascent for pregnancy and lactation. For example, a cross‐gestation PBPK model has been developed to describe the tail‐phase pharmacokinetics of LA‐CAB/RPV following discontinuation early in pregnancy, but such models require broader validation and application across compounds.^22^ Similarly, PBPK approaches have been used to compare potential fetal exposure with daily oral vs. LAI antipsychotics (e.g., olanzapine and aripiprazole), underscoring the need to understand formulation‐specific kinetics during pregnancy.^23^ Although lactation PBPK models exist for daily oral drugs and guidance is available,^24^, ^25^, ^26^, ^27^ adaptation to long‐acting products and systematic validation are still lacking. Collectively, these gaps limit confident decision‐making on initiation, continuation, or discontinuation of long‐acting therapies during pregnancy and breastfeeding. While global under‐investment in maternal and pediatric research represents a broad, systemic funding gap, sustainable local and regional research funding is needed to provide complementary, context‐specific support for operational and implementation studies, helping stabilize research ecosystems in settings where maternal and pediatric needs are greatest.

#### Critical actions

Conduct targeted clinical studies to characterize changes in the pharmacokinetics of long‐acting therapeutics across pregnancy and postpartum, including maternal and newborn sampling at delivery to quantify late‐gestation fetal exposure and breastmilk sampling to estimate infant exposure during lactation. Leverage PBPK modeling—across gestation and lactation—to bridge evidence gaps, guide dose/exposure targets, and inform decisions on initiation, continuation, or discontinuation of therapy, with adaptation and validation for long‐acting formulations. Prioritize compounds with established pregnancy safety to enable accelerated pathways (consistent with a tiered approach).^16^ Advance validated, human pregnancy‐relevant NAMs (e.g., gastruloids, placenta‐on‐chip) to strengthen key components of DART assessments, supported by enabling policies, dedicated funding, and precompetitive validation.^28^, ^29^, ^30^ These actions will improve the predictive value of preclinical evidence and facilitate ethical inclusion of pregnant and breastfeeding women in clinical trials.

#### Enabling partnerships

The Community of Practice advocate that global health research funders should highlight some of the gaps identified above as areas of interest under relevant funding schemes. Industry study sponsors and research networks with interest in medication use during pregnancy and breastfeeding should use their existing infrastructure and expertise to develop and implement protocols to addressing these gaps. There is an opportunity for regulatory agencies like the US Food and Drug Administration (FDA), European Medicines Agency (EMA), the African Medicines Agency (AMA), the UK Medicines and Healthcare products Regulatory Agency (MHRA), Japan Pharmaceuticals and Medical Devices Agency, and China National Medical Products Administration to work with PBPK modelers to advance pregnancy and lactation models and to develop a framework for expanding their application in the long‐acting therapeutics space. In the US, recent changes in NIH policy on non‐animal research,^31^ and associated cross‐agency initiatives at the FDA, serve as exemplars for matching policy with action^32^ to drive progress in this area. The use of NAMs for DART studies requires a special focus, and multistakeholder groups like the Community of Practice can contribute to draft regulatory guidance and public consultations to inform future policies.

### Pediatric health

#### Existing gaps

There is a limited understanding of the pharmacokinetics and pharmacodynamics of many drugs in children, **i**n the setting of rapid developmental changes in body size, composition, and physiological function of children from birth through adolescence.^33^ This is partly attributable to the exclusion of children from clinical trials of new drugs due to the lack of appropriate age‐appropriate preclinical models to inform early‐stage pediatric clinical trials, complexities of conducting clinical trials in children (e.g., diversity of age groups, need for age‐appropriate formulations), and the dynamic nature of pediatric physiology (**Figure** **1**).^34^ Efforts to prioritize diseases beyond HIV that afflict pediatric populations (especially younger children) beyond HIV face significant uncertainty, especially regarding high‐burden epidemics such as respiratory syncytial virus and malaria.^35^, ^36^ Additionally, there remains ambiguity and inconsistency among regulatory bodies regarding the acceptance and qualification of modeling and simulation approaches.^37^ Recent global initiatives have led to commendable strides, notably through the WHO's GAP‐f program, which aims to accelerate the development and equitable delivery of child‐friendly medicines across high‐burden diseases and underserved populations.^38^ Regulatory frameworks such as the US FDA's Pediatric Research Equity Act and Best Pharmaceuticals for Children Act have incentivized pediatric drug development and clarified ethical standards for trial conduct.^39^ Despite these advances, persistent gaps remain: children—especially those under 9 years old and in low‐resource settings—are still underrepresented in trials, and many treatments lack age‐specific safety and efficacy data.^40^ Ethical complexities, limited pediatric‐friendly, palatable formulation options, and logistical barriers all contribute to hindering trial feasibility. Only a few long‐acting therapeutics are currently approved for use in children, primarily for managing endocrine disorders, including triptorelin and leuprolide acetate for central precocious puberty.^41^, ^42^ There is a need for robust PK, safety, and efficacy data for long‐acting therapeutics used for other indications that affect children. Moreover, the adoption of adaptive designs and real‐world data remains slow, despite their potential to enhance pediatric trial efficiency and relevance.^43^

#### Critical actions

To address these gaps, policies should focus on creating inclusive clinical trial designs, maintaining long‐term investment in pediatric‐specific drug formulations, and fostering coordinated international efforts to guarantee equitable access to medicines for all children. Given the dynamic nature of pediatric physiology, establishing age‐appropriate criteria for what constitutes “long‐acting” is critical. Investment in innovative technologies—such as microarray patches designed for safe, convenient use in young children—will address a major gap in pediatric drug delivery and enhance treatment accessibility and compliance. Integrating PBPK model‐informed decisions into both the development of long‐acting pediatric formulations and their clinical trial design is essential. Such models are needed to guide strategies for technologies like microneedle array patches—as for models already developed for adults^44^, ^45^, ^46^—and to assess the age‐appropriateness of existing LAIs. Early and structured engagement with regulatory authorities, including pre‐submission meetings, is essential. Establishing a global framework to align existing pediatric medicines with suitable long‐acting drug delivery systems would serve as a powerful catalyst for progress in this field.

#### Enabling partnerships

Achieving these will require multifaceted, cross‐stakeholder partnerships that bring together academic researchers, clinical trialists, pharmaceutical innovators, and technology developers to advance age‐appropriate long‐acting formulations and PBPK modeling approaches. Collaboration with regulatory authorities is essential for early engagement and streamlined approval pathways, while global health organizations and policymakers must coordinate efforts to ensure equitable access and harmonized standards. Industry partners specializing in novel delivery systems—such as microneedle and microarray technologies—should work alongside clinical trial networks to design inclusive, adaptive studies. Finally, international consortia and funding bodies will play a pivotal role in sustaining investment and creating a global framework that aligns existing pediatric medicines with innovative long‐acting platforms. The Community of Practice will continue to facilitate cross‐learning and knowledge synthesis as evidence and enabling technologies evolve.

### Access considerations

#### Existing gaps

Recent voluntary licensing agreements for LA‐CAB/RPV and lenacapavir are transformational in facilitating equitable access to long‐acting antiretrovirals. A few additional indication‐agnostic barriers remain for timely access in LMICs. These licenses typically exclude technology transfer, leaving manufacturers without critical formulation and process know‐how for complex LAIs (**Figure** **1**).^10^ For instance, LA‐CAB/RPV production requires expertise in sterile nanosuspension and depot technologies, while lenacapavir involves specialized subcutaneous injection and stability processes. These technologies and associated manufacturing know‐how are not widely accessible to generic manufacturers because the underlying formulation may rely on proprietary processes and product‐specific design elements that are still under patent protection.^47^

The absence of structured tech transfer may prolong reverse‐engineering efforts, delaying regulatory filings, and WHO prequalification, with generic LA‐CAB/RPV unlikely before 2027. Furthermore, cold‐chain infrastructure, health system capacity, high costs, and trained personnel for administration may compound implementation challenges, especially in rural settings with inadequate infrastructure.^48^, ^49^ Similarly, while several generic manufacturers have been selected to produce lenacapavir under direct licensing arrangements with Gilead and will build capacity to supply millions of doses across more than 120 low‐ and lower‐middle‐income countries, the long‐term impact of these efforts still depends on overcoming technical complexity and scaling up sterile injectable manufacturing. Similarly, exclusion of many upper middle‐income countries under this model may not deliver an inclusive licensing and pricing strategy that will ensure widespread availability for a truly global impact.^50^ Service delivery models remain fragmented across many LMICs, particularly within maternal and child healthcare. This fragmentation is evident in the lack of continuity and harmonization of services for conditions such as gestational diabetes, which has long‐term implications for both maternal and child outcomes.^51^ Because injectable therapies are often perceived as treatments for severe illness (e.g., patients prescribed LAI antipsychotics had greater illness severity/chronicity vs. oral),^52^ it is unclear how this perception may shape the acceptability and uptake of LAI in pregnancy and pediatric populations.

#### Critical actions

Proactive measures to share proprietary know‐how and strengthen manufacturing capacity are needed to ensure voluntary licensing leads to equitable, timely access. Innovators must address affordability and sustainability as part of their development programs, ensuring innovations are not limited to wealthy populations. Equitable access and affordability of long‐acting therapeutics through local manufacturing and accessible funding models in LMICs must be promoted.^53^ Governments should take ownership of healthcare funding and prioritize inclusion of long‐acting therapeutics in essential medicine lists. Communities and healthcare workers must be engaged early to facilitate context‐specific knowledge sharing and build trust to ensure successful implementation. Uptake and adherence may be adversely affected by the lack of organized methods to include men's viewpoints and assistance during their partners' involvement in research or treatment programs.^54^ Additionally, operational and implementation research is needed to support real‐world delivery and uptake in LMIC countries.^55^ Integrated service delivery models are needed in healthcare systems for achieving optimal maternal and child health outcomes. Such integration would allow seamless coordination of reproductive health services, antenatal care, and treatment or prevention programs—such as LA‐CAB or lenacapavir for HIV PrEP—while facilitating informed decisions about pregnancy planning and whether to initiate, continue, or discontinue long‐acting therapeutics.

#### Enabling partnerships

Achieving timely and equitable access to long‐acting therapeutics in LMICs requires coordinated, multisector partnerships. Structured technology transfer and capacity building between originators, public health organization (e.g., the Medicines Patent Pool), and LMIC manufacturers are essential to overcome manufacturing barriers. Affordability must be addressed through collaborations involving global health stakeholders (e.g., Unitaid, CHAI) and global financing mechanisms to secure pooled procurement and donor‐backed guarantees. Policy integration requires engagement of WHO, ministries of health, and technical partners to ensure inclusion in essential medicine lists. Community trust‐building should leverage partnerships with relevant organizations and local advisory structures to deliver culturally tailored strategies. Operational research requires collaboration among global research networks focused on maternal and pediatric health (e.g., the International Maternal Pediatric Adolescent AIDS Clinical Trials [IMPAACT], healthcare and academic institutions to generate real‐world evidence for LMIC contexts). Finally, partnerships between health system integration and capacity building organizations and national health systems actors are needed to align reproductive, antenatal, and therapeutic services for better outcomes. It is important to foster inclusive representation across various regions, especially underrepresented Francophone African countries. The Community of Practice will continue to facilitate activities that amplify diverse voices and promote equitable access.

### Regulatory and Ethics

#### Existing gaps

Across regions, clinical data for vulnerable populations remain limited, creating uncertainty for regulators and slowing decision‐making.^56^, ^57^ Enrolling children in clinical research continues to involve complex ethical considerations, including consent and assent processes, acceptable risk thresholds, and safeguards for minors in both treatment and prevention trials. Additionally, inconsistent pediatric regulatory requirements across jurisdictions—such as differences in age thresholds, mandated study designs, and data expectations—complicate global development programs and contribute to delays in recruitment and approval. There is also a need for long‐term pharmacovigilance and follow‐up data for maternal, fetal, and childhood exposure to long‐acting therapeutics (**Figure** **1**).^15^ Together, these systemic gaps limit the ability to generate robust evidence and hinder equitable access to long‐acting products for maternal and pediatric populations.

#### Critical actions

An immediate gap that needs attention is the development of pregnancy drug exposure registries that facilitate longitudinal monitoring of maternal and child outcomes from birth to early (e.g., the Danish Diabetes Birth Registry 2)^58^ (p. 2) and late childhood. Documentation of formulation types and dosing frequencies will improve the utility of datasets from such registries. Periodic communication and collaboration between regulators, researchers, and patient advocacy communities can align expectations and support guidelines development.^59^ Further, regulators need to improve cross‐agency transparency and knowledge sharing, harmonize pathways where possible, and strengthen existing regulatory processes for expedited review of novel therapeutics for maternal and pediatric health indications.^60^ Efforts should be made to harmonize regulatory requirements across regions to streamline approval processes and facilitate global access.

#### Enabling partnerships

The US FDA, EMA, and UK MHRA maintain strong collaborative ties through initiatives that advance regulatory convergence and reliance. These agencies share scientific advice, conduct joint inspections, and exchange information under confidentiality agreements to harmonize standards and minimize duplication of effort. A similar framework exists among the Association of Southeast Asian Nations (ASEAN) member states, including multiple harmonization mechanisms and the ASEAN Joint Assessment Procedure. In a similar vein, the AMA—established in 2019 and operational since 2021—was created to accelerate drug approval across Africa by harmonizing regulatory standards, reducing duplication, and establishing a unified evaluation framework. AMA promotes reliance mechanisms such as joint assessments and the Continental List of Human Medicinal Products, enabling national authorities to adopt shared reviews rather than conducting full independent assessments. AMA also supports fast‐track and emergency‐use pathways for priority products, strengthens cross‐agency transparency, and builds regulatory capacity through partnerships with WHO, Africa CDC, and AUDA‐NEPAD. These efforts streamline approval processes, improve efficiency, and facilitate timely access to essential medicines across the continent. Such partnerships will remain critical to driving cross‐agency transparency, fostering mutual understanding of review pathways, and creating opportunities for harmonization of regulatory requirements. A multistakeholder approach could further enable the establishment of global interoperable pregnancy exposure registries with harmonized data standards. Clinical researchers must collaborate with patients, communities, and advocacy groups to co‐develop research protocols that align with community priorities and build trust. Similarly, partnerships between regulatory agencies, global health organizations, and implementation actors will provide opportunities to harmonize requirements for timely, equitable access to long‐acting therapeutics for maternal and pediatric health indications. The Community of Practice will continue to engage stakeholders in ethics and regulatory sectors worldwide and disseminate lessons learned from these engagements.

The regulatory, ethics, and access domains are cross‐cutting, influencing both maternal and pediatric health. This interdependence underscores the need for coordinated, multisector approaches to accelerate the development, evaluation, and equitable implementation of long‐acting therapeutics (**Figure** **1**).

## SUMMARY OF RECOMMENDATIONS

Despite the gaps identified above, investing in long‐acting therapeutics offers several advantages for innovators and manufacturers. For maternal and pediatric health, they have the potential to address a uniquely high‐impact, high‐burden set of conditions in which even modest improvements in coverage, adherence, or product stability translate into population‐level health gains, large cost savings, and substantial global health market pull. Additionally, as long‐acting delivery steadily becomes a preferred—and in some therapeutic areas, expected—standard of care, manufacturers who fail to develop long‐acting options may face a future market in which they are effectively noncompetitive.

**Figure 2:**
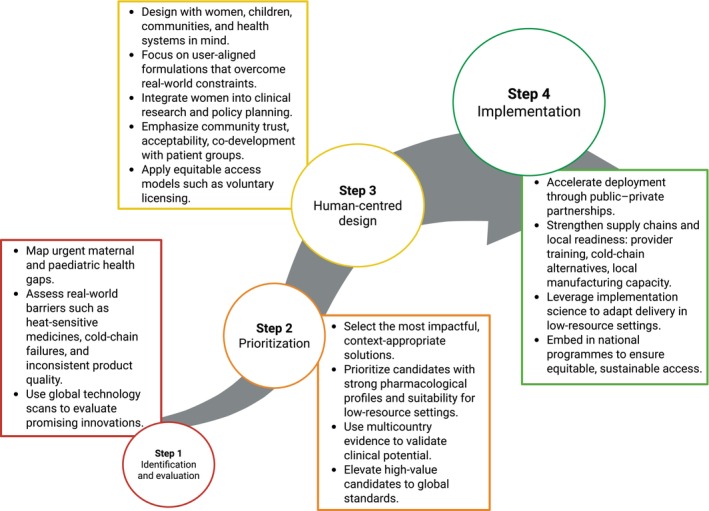
Roadmap for advancing long‐acting therapeutics for maternal and pediatric health. This roadmap outlines four interconnected steps for accelerating the development, evaluation, and equitable delivery of long‐acting therapeutics. (1) Identification and evaluation of gaps: defines priority maternal and pediatric health needs and evidence gaps. (2) Prioritization: highlights the most impactful opportunities for innovation and resource allocation. (3) Human‐centered approach: ensures that product development, trial design, and policy decisions are shaped by lived experience, community input, and real‐world constraints. (4) Implementation: focuses on coordinated regulatory, access, and health‐system strategies that enable scalable, equitable adoption of long‐acting therapeutics across diverse settings.

We present a clear roadmap for realizing the full potential of long‐acting therapeutics in these populations (**Figure** **2**). As highlighted in our maternal health session review in this issue, this approach led to the successful uptake of carbetocin for postpartum hemorrhage and ongoing progress in efforts to scale up access to lenacapavir for HIV prevention.^18^ Clearly, it requires coordinated actions across the entire continuum of drug development by all stakeholders. To address maternal health gaps in the use of long‐acting therapeutics, targeted PK studies across pregnancy and postpartum are essential, including maternal and newborn plasma sampling at delivery and breastmilk sampling after delivery to assess fetal exposure. PBPK modeling should be leveraged to bridge evidence gaps for pregnancy and lactation, while prioritizing compounds with established safety profiles in pregnancy to enable streamlined regulatory pathways. Development and validation of human‐relevant NAMs for preclinical DART studies must be accelerated, supported by dedicated funding and precompetitive collaborations. Regulatory agencies should collaborate with PBPK modelers to advance pregnancy and lactation frameworks for long‐acting formulations. Multistakeholder partnerships—including industry sponsors, global health funders, and research networks—are critical to implement inclusive protocols and ensure sustainable, context‐specific research capacity in LMICs.

To prevent hitherto persistent gaps in pediatric drug development from extending to long‐acting therapeutics, stakeholders should prioritize inclusive and adaptive trial designs that reflect the diversity of age groups and physiological dynamics in children. Long‐term investment in age‐appropriate formulations and innovative delivery systems will be critical to improving accessibility and acceptability. PBPK and popPK modeling should be integrated into formulation development and trial design to optimize dosing strategies. Early engagement with regulatory authorities must be strengthened to ensure alignment with expectations and accelerate approvals. Establishing a global framework that matches existing pediatric medicines with suitable long‐acting technology platforms, supported by coordinated international partnerships and sustained funding, will ensure equitable access and catalyze progress across multiple pediatric indications. Co‐development of research protocols with patient groups and community representatives is an important way to build trust within communities.

To ensure timely and equitable access to long‐acting therapeutics in LMICs, originator companies should implement structured technology transfer and capacity building programs to overcome manufacturing barriers for complex injectables. Governments must prioritize inclusion of these therapeutics in essential medicine lists and allocate sustainable funding, while global health partners should support affordability through pooled procurement and donor‐backed guarantees. Early engagement of communities and healthcare workers is critical to address stigma, build trust and identify training needs, alongside gender‐inclusive strategies that incorporate men's perspectives. Linkage of reproductive health and antenatal care with treatment and prevention programs should be promoted to improve maternal and child health outcomes. Finally, operational research and multisector partnerships—including innovators, LMIC manufacturers, WHO, funders, and research networks—are essential to generate observational evidence and accelerate implementation. Prioritizing supply chain resilience, including buffer stock, cold chain solutions, and provider training hubs, will further strengthen delivery systems.^61^ Developing and fostering long‐acting therapeutics that do not require a cold chain will be considerably advantageous in LMIC settings. Governments can leverage WHO's Networks of Care to integrate long‐acting therapeutics into maternal, newborn, and child health services at every level of the health system.^61^ Strengthening health systems—including workforce training, infrastructure, and commodity supply—is essential to sustain equitable delivery. Cross‐ministerial financing mechanisms should be established to protect maternal and child health programs from fluctuations in donor funding.^62^

To address regulatory and ethical gaps in maternal and pediatric drug development, stakeholders should prioritize establishing global pregnancy exposure registries with harmonized data standards to enable longitudinal monitoring from birth through childhood. These registries should capture formulation types and dosing frequencies to enhance data utility. Regulators must strengthen cross‐agency transparency, harmonize requirements across regions where possible, and expand reliance mechanisms to accelerate review of novel long‐acting therapeutics. Leveraging existing collaborative frameworks between regulatory agencies designed to streamline approval processes, reduce duplication, and facilitate timely access will facilitate equitable access to long‐acting therapeutics for vulnerable populations. It is critical for innovators to understand how these mechanisms apply to long‐acting therapeutics for maternal and pediatric health indications.

## FUNDING

This work was supported by Unitaid (grant number 2020‐38‐LONGEVITY). An.O. and Ad.O. acknowledge support from the US National Institutes of Health (NIH) through LEAP award (2R24AI118397–11). Ad.O. also acknowledges funding from Wellcome (227288/Z/23/Z). E.D.W. receives funding from the United States NIH through a mentored career development award, under award number K23AI150349. The views expressed in this White Paper are those of the authors and not necessarily those of the funders.

## CONFLICTS OF INTEREST

An.O. is a director of Extentus Pharma Ltd. and co‐inventor of drug delivery patents. An.O. has been co‐investigator on funding received by the University of Liverpool or Extentus Pharma Ltd. from ViiV Healthcare, Bicycle Therapeutics, and Gilead Sciences and has received personal fees from Gilead, Shionogi, and Assembly Biosciences. Other authors declare no conflicts of interest relevant to the content of this White Paper.

## Supporting information


Table S1
