# Supplementary material for: A White Paper on Advancing Long‐Acting Therapeutics for Maternal and Pediatric Health by Bridging Gaps in Clinical Research, Access and Regulation
Source: Clin Pharmacol Ther. 2026 Apr 14:10.1002/cpt.70288. Online ahead of print. doi: 10.1002/cpt.70288 (PMC13339518; doi:10.1002/cpt.70288)
Supplement: Supplementary file 1 — Table S1 [file CPT-9999-0-s001.docx]

**Table S1.** Members of the Community of Practice for Long-acting Therapeutics for Maternal and Paediatric Health who participated in the 1^st^ July 2025 workshop.

| **Member** | **Affiliation** | **Area of expertise (sector)** |
| --- | --- | --- |
| Rana Abutaima | Zarqa University, Jordan | Pharmacologist (academic) |
| Dorothy Akongo | Busoga Health Forum, Uganda | Public Health (NGO) |
| Abdulnaser Alsharaa | PharmaPrimes Laboratories, Jordan | Bioanalysis (industry) |
| Moherndran Archary | Africa Health Research Institute, South Africa | Pediatric infectious diseases (academic/clinical) |
| Shakir Atoyebi | University of Liverpool, UK | Pharmacometrics (academic) |
| Benoit Bestgen | Medicines for Malaria Venture, Switzerland | Product development partnership (NGO) |
| Robert Bies | University of Buffalo, USA | Pharmaceutical sciences (academic) |
| Osei Boateng | OKB Foundation, USA | Global health access (NGO) |
| Andrew Butler | Medicines and Healthcare products Regulatory Agency, UK | Clinical pharmacology (regulatory) |
| Edmund Capparelli | University of California, San Diego, USA | Pediatric pharmacology (academic) |
| Rachel Daley | University of Liverpool, UK | Stakeholder engagement (academic) |
| Layla Davies | University of Liverpool, UK | Global health access (academic) |
| Joelle Dountio | Treatment Action Group, USA | Global health (NGO) |
| Henry Enzama | Makerere University, Uganda | Pharmacology (academic) |
| Pierre Gashema | Repolicy Research Centre, Rwanda | Global health (academic) |
| Katila George | Queen Mary University Hospital London, UK | Clinical trials (clinical) |
| Dan Hawcutt | University of Liverpool / Alder Hey, UK | Pediatric pharmacology (academic/clinical) |
| Rene Holm | University of Southern Denmark | Chemistry and pharmacy (academic) |
| Patrick Gad Iradukunda | Rwanda Food and Drugs Authority | Quality and compliance (academic/regulator) |
| Elodie Jambert | Medicines for Malaria Venture, Switzerland | Access and product management (NGO) |
| Mili Karina | Nurturing Moms, Kenya | Maternal health (clinician) |
| Linda Lewis | Clinton Health Access Initiative, USA | Clinical and regulatory (NGO) |
| Grace Miheso | Independent Consultant, Kenya | Global health (community) |
| Sebastien Morin | Medicines Patent Pool, Switzerland | Global public health (NGO) |
| Andrew Morrison | University of Liverpool, UK | Partnerships (academic) |
| Sharon Nachman | Stony Brook, USA, and the IMPAACT Network | Pediatrics (academic) |
| Shadia Nakalema | Infectious Diseases Institute, Uganda | Clinical trials (academic) |
| Emily Njunga | PATH, Kenya | Maternal and newborn health (NGO) |
| Nathaniel Nkrumah | Ghana Food and Drugs Authority, Ghana | Infectious diseases (regulator) |
| Carolyne Odula-Obonyo | The University of Nairobi Health Services, Kenya | Obstetrics and gynaecology (clinical) |
| Brenda Okware | Gilead, Ireland | Clinical development (industry) |
| Adeniyi Olagunju | University of Liverpool, UK | Perinatal pharmacology (academic) |
| Andrew Owen | University of Liverpool, UK | Pharmacology (academic) |
| Martina Penazzato | WHO, Switzerland | Public health (NGO) |
| Natella Rakhmanina | Children’s National Medical Center, USA | Pediatrics (clinician) |
| Mphako Brighton Ratlabyana | South African Health Products Regulatory Authority, South Africa | Pharmaceutical evaluations (regulator) |
| Rachel Scott | Georgetown University, USA | Obstetrics and gynaecology (clinical/academic) |
| Kimberly Struble | Food and Drug Administration, USA | Regulatory |
| Andre-Marie Tchouatieu | Medicines for Malaria Venture, Switzerland | Product development partnerships (NGO) |
| Catherine Unsworth | University of Liverpool, UK | Chemistry (academic) |
| Prajith Venkatasubramanian | University of Liverpool, UK | Pharmacologist (academic) |
| Catriona Waitt | University of Liverpool, UK | Clinical pharmacology and global health (academic) |
| Ethel Weld | Johns Hopkins University School of Medicine, USA | Pharmacology (academic) |
| Janine Winterbottom | The Walton Centre NHS Foundation Trust, UK | Maternal health (clinical) |
| Leena Zino | Dada Consultancy | Regulatory affairs (industry) |

Abbreviations: NGO, non-governmental organization.
